# Effect of substitution on the superconducting phase of transition metal dichalcogenide Nb(Se$_{x}$S$_{1-x}$)$_{2}$ van der Waals layered structure

**DOI:** 10.1038/s41598-021-94000-2

**Published:** 2021-07-26

**Authors:** Prutthipong Tsuppayakorn-aek, Prayoonsak Pluengphon, Piya Phansuke, Burapat Inceesungvorn, Wutthikrai Busayaporn, Pungtip Kaewtubtim, Thiti Bovornratanaraks

**Affiliations:** 1 Extreme Conditions Physics Research Laboratory (ECPRL) and Physics of Energy Materials Research Unit, Department of Physics, Faculty of Science, Chulalongkorn University, Bangkok, 10330 Thailand; 2 Thailand Centre of Excellence in Physics, Ministry of Higher Education, Science, Research and Innovation, 328 Si Ayutthaya Road, Bangkok, 10400 Thailand; 3 Division of Physical Science, Faculty of Science and Technology, Huachiew Chalermprakiet University, Samutprakarn, 10540 Thailand; 4 Department of Science, Faculty of Science and Technology, Prince of Songkla University, Pattani Campus, Pattani, 94000 Thailand; 5 Department of Chemistry, Center of Excellence in Materials Science and Technology and Materials Science Research Centre, Faculty of Science, Chiang Mai University, Chiang Mai, 50200 Thailand; 6 Synchrotron Light Research Institute (Public Organization), Nakhon Ratchasima, 30000 Thailand

**Keywords:** Condensed-matter physics, Physics, Materials science, Condensed-matter physics, Theory and computation

## Abstract

By means of first-principles cluster expansion, anisotropic superconductivity in the transition metal dichalcogenide Nb(Se$_{x}$S$_{1-x}$)$_{2}$ forming a van der Waals (vdW) layered structure is observed theoretically. We show that the Nb(Se$_{0.5}$S$_{0.5}$)$_{2}$ vdW-layered structure exhibits minimum ground-state energy. The Pnnm structure is more thermodynamically stable when compared to the 2H–NbSe$_{2}$ and 2H–NbS$_{2}$ structures. The characteristics of its phonon dispersions confirm its dynamical stability. According to electronic properties, i.e., electronic band structure, density of states, and Fermi surface indicate metallicity of Nb(Se$_{0.5}$S$_{0.5}$)$_{2}$. The corresponding superconductivity is then investigated through the Eliashberg spectral function, which gives rise to a superconducting transition temperature of 14.5 K. This proposes a remarkable improvement of superconductivity in this transition metal dichalcogenide.

## Introduction

Transition metal dichalcogenides (TMDs) in the form of layered structures^1–4^ have recently attracted much attention due to their fascinating superconductivity mechanisms. Charge-density waves (CDWs) transition, usually coexisting with Cooper pair condensation in TMDs, was previously reported to have been associated with structural distortions of the lattice sites^5–8^. For instance, CDW effect was found to promote superconductivity in 1T-TaS$_{2}$ structure^9,10^. Following this, pressure significantly affects superconductivity in this very structure as well: a pressure range of 3-25 GPa gives rise to a superconducting transition temperature (T$_{c}$) of 5 K. Moreover, there are also studies that give more detailed discussions on the layered structures of TMDs at both ambient and high pressure, for example, WTe$_{2}$ was confirmed to superconduct^11^ and clearly shown that the van der Waals (vdW) forces play an important role^12^.

More recently, the vdW forces holding the two-dimensional layers collectively in NbSe$_{2}$ bulk were shown to be the case^2^ in which CDW directly related to Fermi surface in the 2H-NbSe$_{2}$ structure. To the best of our knowledge, it was well-known that a key factor for the high-T$_{c}$ superconductivity depends upon the large Fermi surface topology (FST) size. Also, CDW was found to improve superconductivity in such an electron-phonon coupled system of the 2H-NbSe$_{2}$ structure^13^. Subsequently, a theoretical study revealed that d-electrons of Nb predominantly influence the value of T$_{c}$^14^. The other explanation implies that in order to improve superconductivity in NbSe$_{2}$, the electronic topological (ETT) of Nb should be investigated^15^. The concept of ETT, as also shown to be associated with FST, has been used to elucidate the nature of electronic structures of this material. As a result, the relationship between ETT and FST now provides new insight into the nature of superconductivity in TMDs: the effect of ETT on Nb causes T$_{c}$ to vary. In addition to this, 4d-electron of Nb somehow couple with 4p-electron of Se to form a Cooper pair, which in turn significantly promotes T$_{c}$ in 2H-NbSe$_{2}$^16^. Furthermore, experimental observation has pointed out that there is an interplay between CDW, FST, and superconductivity for the anisotropy in the electron-phonon coupling (EPC) and Fermi velocities that the EPC and multiband structure of the FST are crucial for superconductivity^17^.

Heil et al.^17^investigated superconductivity in the 2H-NbS$_{2}$ structure using the *ab initio* anisotropic Migdal-Eliashberg (ME) theory and found that superconductivity is associated with the FS topology, exhibiting an unusually strong EPC resulting in the highest T$_{c}$ of approximately 15.3 K. Experimental and theoretical studies on superconductivity in layered quasi-two-dimensional 2H-NbS$_{2}$ and 2H-NbSe$_{2}$ structures have also been conducted^18^. According to the local magnetic field, T$_{c}$ accounting for both the 2H-NbS$_{2}$ and 2H-NbSe$_{2}$ structures reported to be approximately 5.6 K and 7.2 K, respectively. At this point, the FST calculation is a key factor for superconductivity, which demonstrates a positive correlation between T$_{c}$ and pressure. Moreover, superconductivity in the T$_{d}$-MoTe$_{2}$ structure can be observed. The corresponding T$_{c}$ was reported to be 0.1 K, as measured by electrical resistivity measurements^19^. Experimental observations also agree very well with theoretical studies. T$_{c}$ of T$_{d}$-MoTe$_{2}$ is theoretically estimated to be 1.7 K, by employing the role of ME theory and FST^20^. Re-substitutions of Mo are one of the methods for improving T$_{c}$ in MoTe$_{2}$^21^: the substituted Mo$_{0.7}$Re$_{0.3}$Te$_{2}$ gives rise to T$_{c}$ of 4.1 K, compared with the host structure of MoTe$_{2}$ with T$_{c}$ = 0.1 K. Clearly, the atomic-substitution method plays an important role in improving superconductivity in this class of materials which have been demonstrated by both *ab initio* anisotropic ME theory and the FST^17,18,20,21^.

In this work, we aimed to improve the T$_{c}$ of NbSe$_{2}$ by substituting *S*-atom at ambient pressure. We predicted the decoration of Nb(Se$_{x}$S$_{1-x}$)$_{2}$ by a cluster expansion (CE) method. The values of T$_{c}$ are obtained by solving the Allen–Dyne equation^22^ without compression. Regarding the structure of Nb(Se$_{x}$S$_{1-x}$)$_{2}$, we began by determining the ground-state structures of Nb(Se$_{x}$S$_{1-x}$)$_{2}$, where x = 0.5, and compared their energies with those of 2H-NbSe$_{2}$ and 2H-NbS$_{2}$. We also study their electronic properties, i.e., electronic band structure, Fermi surface, and Eliashberg spectral function that are directly related with the superconductivity of the materials.

## Methods

Structural prediction of Nb(Se$_{x}$S$_{1-x}$)$_{2}$ was performed by *ab initio* calculation with the CE method. Random atomic positions, consistent with lattice stability, from atomic substitution obtained from the CE results. The lattice stability leads to predictions of the minimum free energy structures via comparisons of the energy as a function of atomic occupation, as shown in Eq. (1).

1 $$\begin{aligned} E(\sigma ) = \sum _{\sigma }m_\alpha J_\alpha \langle \prod _{i\in \alpha ^{'}} \sigma _{i} \rangle \end{aligned}$$

The energy can be shown in terms of cluster, $\alpha$, by the energy of the CE as a function of occupation. A lattice site, *i*, is associated with an occupation variable $\sigma _{i}$ or a configuration. A set of lattice sites *i* can be represented as a cluster, $\alpha$, which is symmetrically inequivalent. The coefficients, $J _{\alpha }$, give the effective cluster interactions and the multiplicities, $m _{\alpha }$, are symmetrically equivalent to $\alpha$. The sum is taken over all $\alpha$ and the average is taken over all symmetrically equivalent $\alpha ^{'}$. However, the CE energy has not yet been included in DFT calculations. The system was explored by CE^23^, as implemented in the alloy-theoretic automated toolkit^24^ (ATAT) with combined Quantum Espresso (QE) package^25^. A plane-wave energy cutoff of 80 Ry and *k*-point meshes with about 4000 *k*-points was used. The generalized gradient approximation of the Perdew–Burke–Ernzerhof (GGA–PBE) functional^26^ for the exchange-correlation functional was used.

**Figure 1:**
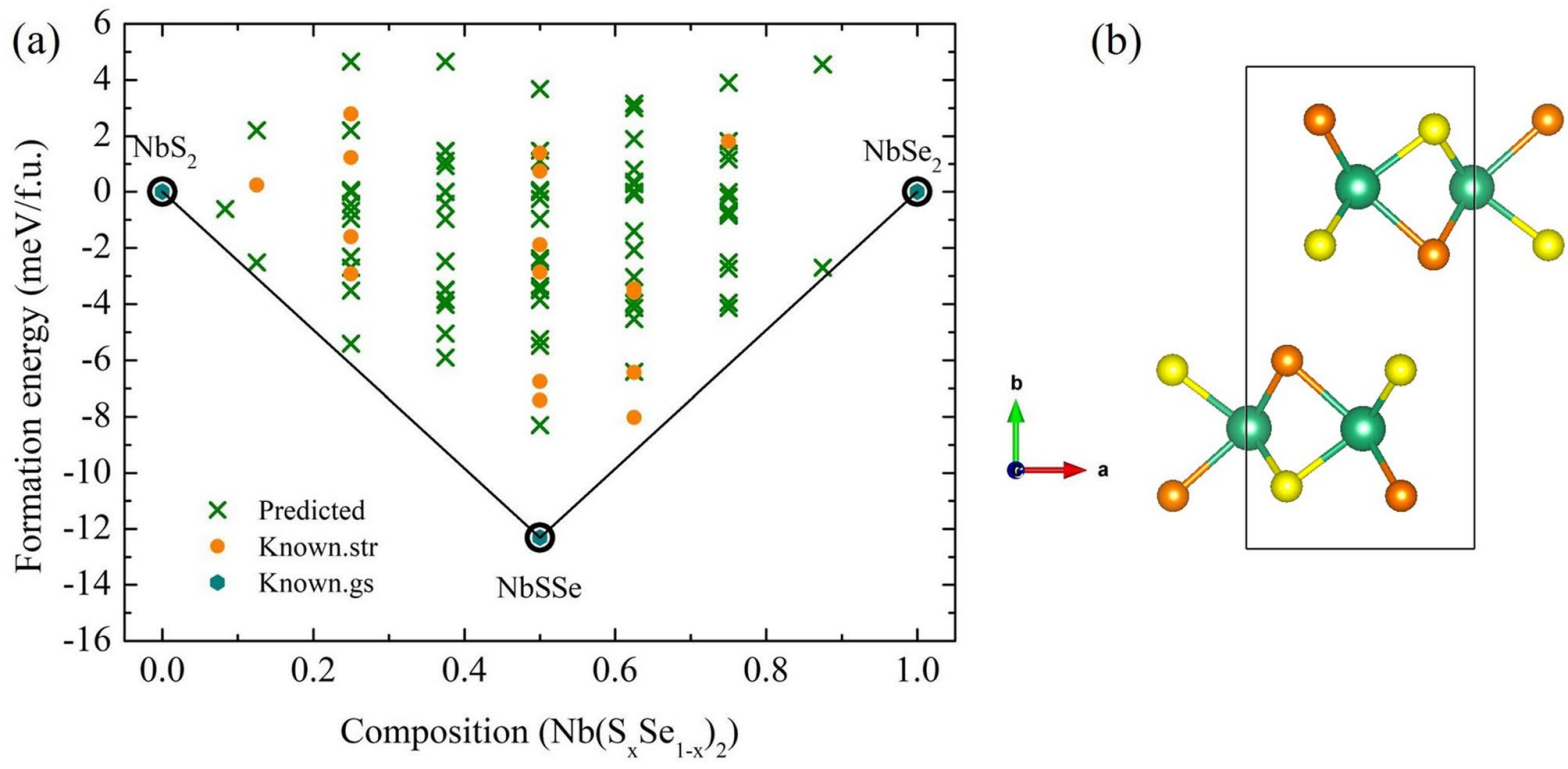
(**a**) The formation energy at ambient pressure. The predicted structures (the cross symbols) refer to structures whose energy has not yet been calculated from DFT, the known str (the circle symbols) refers to structures whose energy has been calculated from DFT, and the known gs (the diamond symbols) refers to the ground states energy that have so far been confirmed by DFT, showing a line on the convex hull. (**b**) The Nb(Se$_{0.5}$S$_{0.5}$)$_{2}$ structure, where the orange sphere, the green sphere and the yellow sphere represent Se, Nb and S, respectively [drawn by VESTA (ver. 3.4.7)^45^ (URL https://jp-minerals.org/vesta/en/download.html)].

The structural optimizations and total energy calculations were performed with GGA-PBE being the exchange-correlation functional. The semi-empirical DFT-D2 vdW corrections^27^, which are the dispersion corrections of the form C$_{6} \cdot$R$^{-6}$, were also treated. The lattice dynamics calculation and the EPC with density functional perturbation theory^28^ were calculated using the QE code^25^. The plane-wave energy cut-off of 80 Ry was selected. Basically, the Eliashberg spectral function depends on the dense k-points; namely, the dense k-points mesh contained all k and k+q grid points, which were covered by the q-points mesh. These conditions of the q-points and the calculated spectral function have been successfully reported in the previous DFT studies^29,30^. Therefore, a 8 $\times$ 4 $\times$ 16 *k*-points and 2 $\times$ 2$\times$ 2 *q*-points computed in the first Brillouin zone (BZ) were used. We calculated T$_{c}$ by solving the approximated Allen–Dyne equation^22^.

2 $$\begin{aligned} T_{c} = \frac{\omega _{log}}{1.2} \exp \Big [ -\frac{1.04(1+\lambda )}{\lambda -\mu ^*(1+0.62\lambda )} \Big ], \end{aligned}$$

where $\omega _{log}$ is the logarithmic average of the spectral function and $\lambda$ is the total EPC strength. The crystal orbital Hamilton population^31^ (COHP) was used to explain the Nb–Se–S chemical bonding, as implemented in LOBSTER code^32^.

## Results and discussion

The thermodynamical stability of the substituted Nb(Se$_{x}$S$_{1-x}$)$_{2}$ reported in Fig. 1a with structures whose energies obtained from the CE method are referred to as the predicted structures (“predicted”), while the energies obtained from DFT calculations denoted by the known structure (“known str”). Finally, the ground-state structures (“known gs”) correspond to those with the lowest possible energies of each proportion. The Nb(Se$_{0.5}$S$_{0.5}$)$_{2}$ structure is the most thermodynamically stable and favored over the 2H–NbS$_{2}$ and 2H–NbSe$_{2}$ structures at ambient pressure (Fig. 1a). The fitting of the effective cluster interactions gives a very accurate cross-validation score of 1 meV/site. Now the orthorhombic structure of Nb(Se$_{0.5}$S$_{0.5}$)$_{2}$ with space group *Pnnm* is undergone geometry optimisation which results in $a=5.8694\,$Å, $b=12.4129\,$Å, and $c=3.3976\,$Åof lattice parameters. Nb atoms located at a 4*g* symmetry site ($-0.515$, $-0.515$, $-0.500$), S atoms at a 4*g* symmetry site ($-0.181$, 0.869, $-0.500$), whereas Se atoms located at a 4*g* symmetry site ($-0.680$, 0.884, $-1.000$), as shown in Fig. 1b.

**Figure 2:**
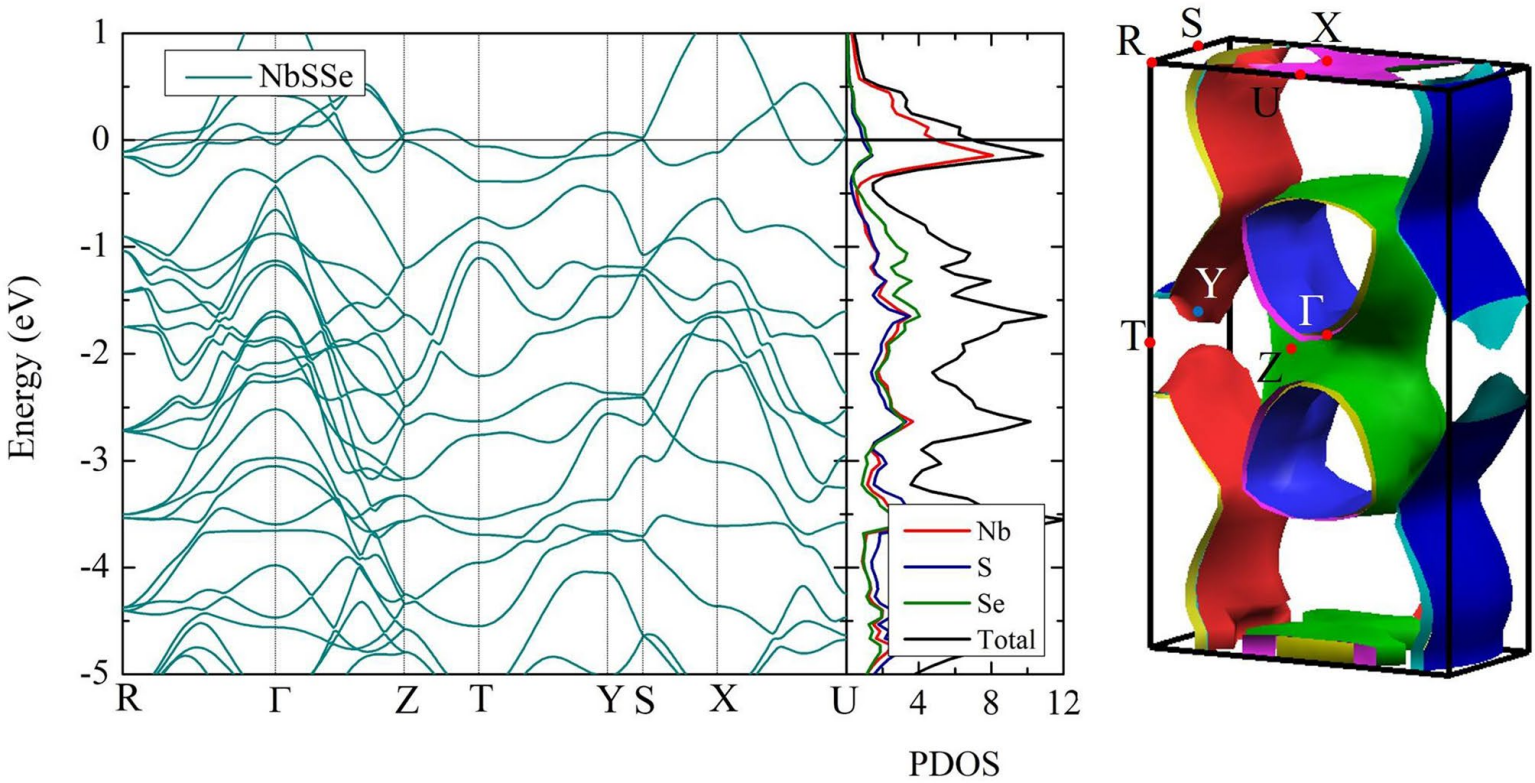
The electronic properties of structure of Nb(Se$_{0.5}$S$_{0.5}$)$_{2}$ (left-hand) the band and DOS, where the red line represents the DOS of Nb atom, the blue line represents the DOS S atom, the green line represents the DOS of Se atom, and the black line represents the total DOS. (right-hand) The FS of the Nb(Se$_{0.5}$S$_{0.5}$)$_{2}$ structure, respectively [drawn by XCrySDen program (ver. 1.5.60)^46^ (URL http://www.xcrysden.org/Download.html#_toc_1)].

Subsequently, the electronic and other physical properties of the Nb(Se$_{0.5}$S$_{0.5}$)$_{2}$ structure are investigated. According to the Nb(Se$_{0.5}$S$_{0.5}$)$_{2}$ structure, one can see that there is a vdW gap between the layers, which requires the adoption of the semi-empirical DFT-D2 vdW corrections^27^. Now as for the electronic band structure and the density of states (DOS), as shown in Fig. 2, it can be seen that the metallicity is confirmed by the electron of the valance band crossing over the Fermi level^33–36^. According to the electronic properties, the Nb(Se$_{0.5}$S$_{0.5}$)$_{2}$ structure is expected to exhibit superconductivity.

**Figure 3:**
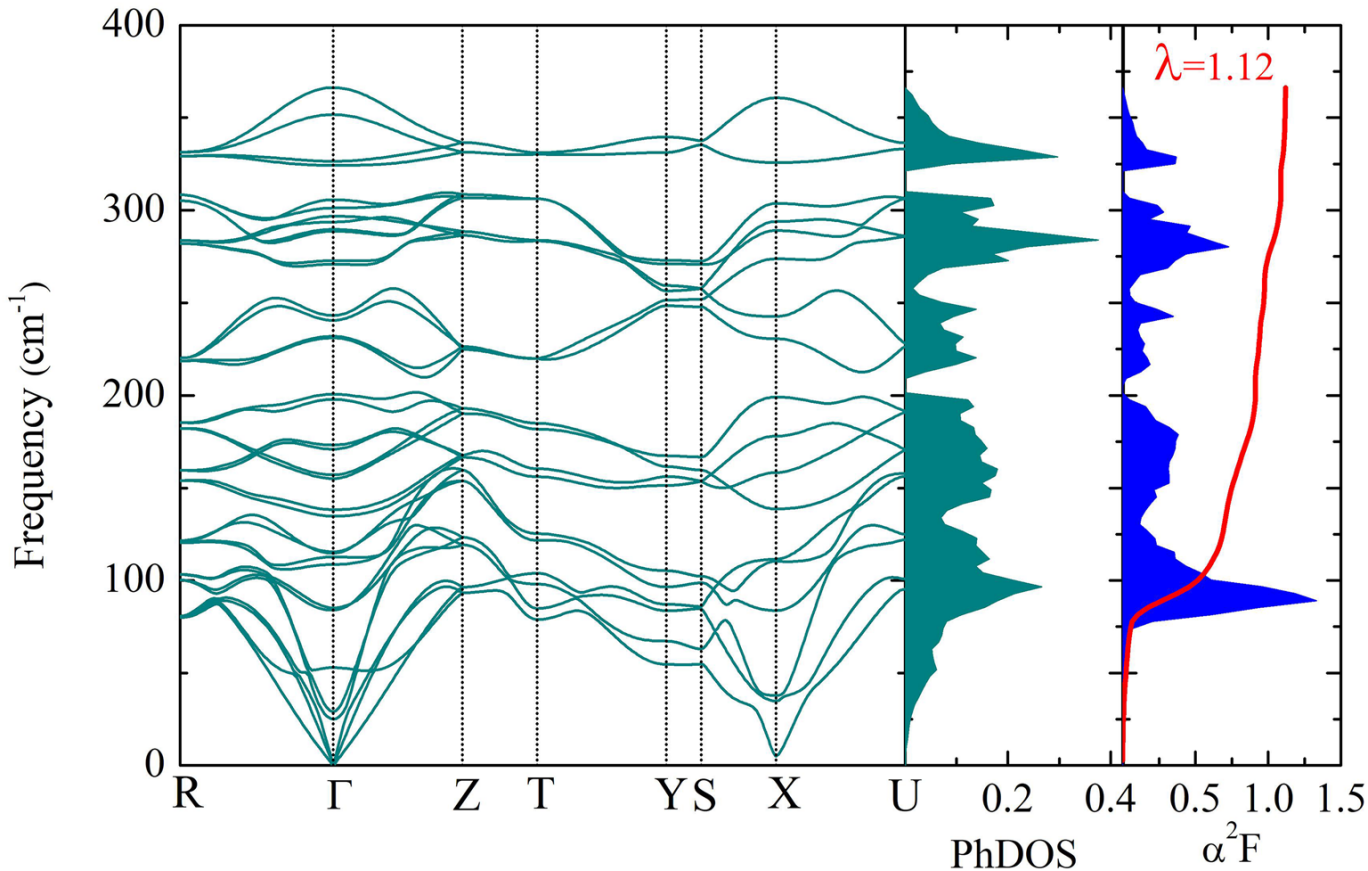
The phonon dispersion of Nb(Se$_{0.5}$S$_{0.5}$)$_{2}$ structure, the phonon density of state of Nb(Se$_{0.5}$S$_{0.5}$)$_{2}$ structure and Eliashberg spectral function of Nb(Se$_{0.5}$S$_{0.5}$)$_{2}$ structure, respectively.

With reference to the stable structure of Nb(Se$_{0.5}$S$_{0.5}$)$_{2}$, we computed the phonon dispersion by means of the linear response method, successfully demonstrating a dynamically stable structure, as presented in Fig. 3. Remarkably, there is a soft-mode at X-point with nearly zero frequency, which suggests that T$_{c}$ of the Nb(Se$_{0.5}$S$_{0.5}$)$_{2}$ structure is likely to significantly increase. Now in order to estimate T$_{c}$, one can solve the Allen–Dynes equation^22^, which requires the Eliashberg spectral function $\alpha ^{2}F$($\omega$) and the integration of lambda $\lambda$ as parameters. As shown in Fig. 3, one can see that $\alpha ^{2}F$($\omega$) is contributed mainly by approximately 0 cm$^{-1}$ to 366 cm$^{-1}$. Following this, $\lambda$ is calculated by integrating the $\alpha ^{2} F$ spectrum. As reported in Fig. 3, $\lambda$ rises slightly in a frequency range 0–75 cm$^{-1}$, then it suddenly shoots at around 100 cm$^{-1}$ and then moderately climbs to the highest frequency. Considering behavior of $\lambda$, it is clear that it reaches a peak of 1.12 ($\omega _{log}$ = 177 K,), when $\mu ^{*}$ is selected to be 0.10. By substituting *S*-atom, the Nb(Se$_{0.5}$S$_{0.5}$)$_{2}$ structure markedly results in the high T$_{c}$ of 14.5 K. At this point, it is interesting to compare the T$_{c}$ of 2H-NbSe$_{2}$ and 2H-NbSe$_{2}$ systems. We also found that the T$_{c}$ of 2H-NbSe$_{2}$ is 7.2 K^37^, while the maximum value of the estimated T$_{c}$ for the NbS$_{2}$ was reported to be 15.3 K by ab initio anisotropic ME theory^17^. Thus, it is interesting to note that the S atom should be considered as the substitution into NbSe$_{2}$. Unfortunately, Nb(Se$_{0.5}$S$_{0.5}$)$_{2}$ does not yet provide the T$_{c}$ value at ambient pressure. Therefore, we proposed the theoretical result of Nb(Se$_{0.5}$S$_{0.5}$)$_{2}$ which may guide further experimental studies.

Now the resulting electronic band structure, DOS, and FST of Nb(Se$_{0.5}$S$_{0.5}$)$_{2}$ are discussed here. We note that the electronic band structure shows a flat band around the FST as well as the presence of the van Hove singularity (vHs) near the Fermi level in DOS. This usually implies the possibility of high $T _{c}$. A plausible explanation for this might be the character of the FST induced by the value of $T_{c}$ in terms of the band structure. The result shows that the FST has a large sheet, which corresponds to the Brillouin zone of the band structure in Fig. 2. Thus, the Nb(Se$_{0.5}$S$_{0.5}$)$_{2}$ can have a higher value of $T _{c}$ when compared with NbSe$_{2}$. Also, we can point out that the hybridization of DOS is important for a stable structure. At this point, this is the hybridization between S and Se atoms at the Fermi level, which indicates that the S substitution is likely to be favored with the Se atom. The solution for the hybridization shows the existence of Nb(Se$_{0.5}$S$_{0.5}$)$_{2}$ but it should be noted that the Nb atom is dominated at the Fermi level when compared to the S atom and the Se atom. This is due partly to the fact that Nb atom enhances the T$_{c}$ of this material.

**Figure 4:**
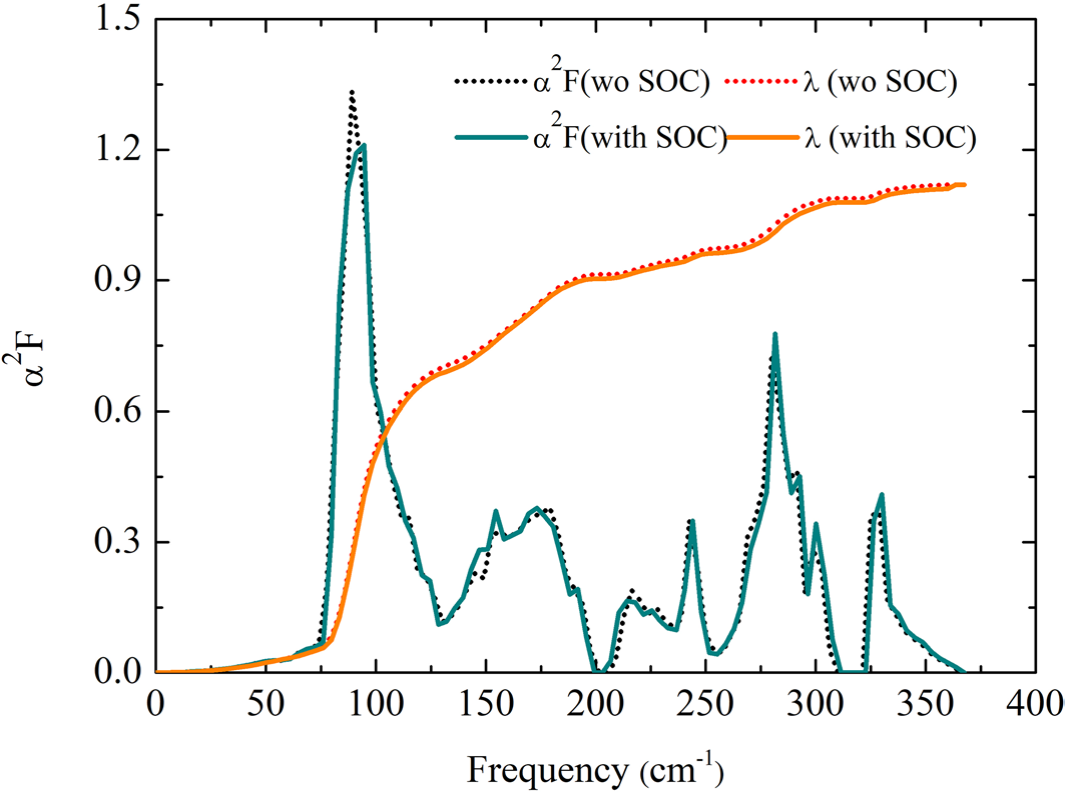
Eliashberg spectral function $\alpha ^{2}F$($\omega$) and the integrating of lambda $\lambda$ in the Nb(Se$_{0.5}$S$_{0.5}$)$_{2}$ structure, where the dotted-black line (the solid-green line) and the dotted-red line (the solid-orange line) represent $\alpha ^{2}F$($\omega$) and $\lambda$ with spin-orbit coupling (without spin-orbit coupling).

Due to such a heavy atom of Nb, spin-orbit coupling (SOC) must be taken into account. As a result, T$$_{c}$$ increases moderately. This is supported by the cases of lead (Pb) and thallium (Tl)^38^, implying that SOC has an influence on the EPC. SOC increases T$$_{c}$$ of Pb but not that of Tl nonetheless. Consequently, it is mandatory to include the effect of the SOC when investigating the Nb(Se$$_{0.5}$$S$$_{0.5}$$)$$_{2}$$ structure. However, the Eliashberg spectral functions with and without the SOC are quite similar (Fig. 4). Similarly, in the case of MoTe$$_{2}$$^39^, it is blatant that including SOC does not affect the Eliashberg spectral function. Moreover, the integration of lambda reaches a peak of 1.12 ($$ _{log}$$ = 170 K,), with $$\mu ^{*}$$= 0.10, and the T$$_{c}$$ with the inclusion of SOC is 14 K. Clearly, the effect of SOC is to some extent negligible in the Nb(Se$$_{0.5}$$S$$_{0.5}$$)$$_{2}$$ structure.

**Figure 5:**
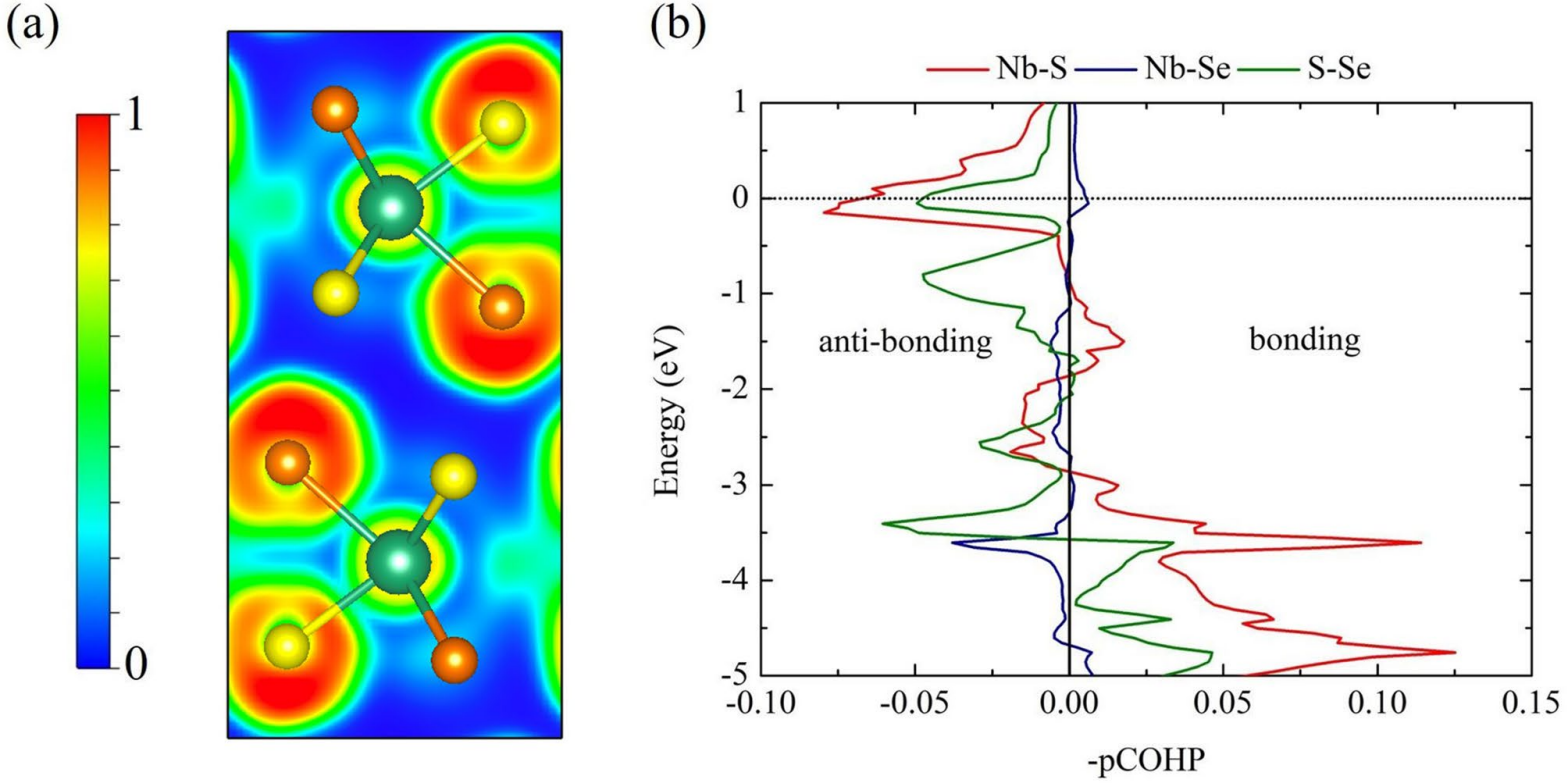
(**a**) The 2D–electron localization function (ELF) in the Nb(Se$_{0.5}$S$_{0.5}$)$_{2}$ structure [drawn by VESTA (ver. 3.4.7)^45^ (URL https://jp-minerals.org/vesta/en/download.html)]. (**b**) Projected crystal orbital Hamilton populations (pCOHPs) in the Nb(Se$_{0.5}$S$_{0.5}$)$_{2}$ structure, where the red line represents the Nb-S pairs, the blue line represents the Nb-Se pairs, and the green line represents the S-Se pairs, respectively.

We further determined the electron localization function (ELF) and the projected crystal orbital Hamilton populations (pCOHP) method^40^, as previously demonstrated on several materials^33–35,41,42^, of Nb(Se$_{0.5}$S$_{0.5}$)$_{2}$. Here, a uniform electron gas of the same density describes the characteristics of the chemical bonding. The ELF surface between Nb and S exhibited weak bonding, while the distribution of electrons between S-Se remained minimal (Fig. 5a). ELF results suffice to accurately describe the nature of the chemical bonding. As shown in Fig. 5b, pCOHP presents both bonding and anti-bonding. This implies that the distribution of electrons plays a significant role in the characteristics of the bonding^36,43,44^. Remarkably, it can be seen that Nb–S and the S–Se pairs interact via anti-bondings, while Nb–Se pair natural bonding. Hence, the high $T _{c}$ value of the Nb(Se$_{0.5}$S$_{0.5}$)$_{2}$ is mainly due to the characteristic of anti-bonding as well as the substitution of S atoms. Finally, it is strongly suggested that the S substitution is likely to improve the $T _{c}$ for other similar materials.

## Conclusion

We found that under ambient conditions, the Nb(Se$_{0.5}$S$_{0.5}$)$_{2}$ structure with space group *Pnnm* is thermodynamically stable compared to the 2H–NbSe$_{2}$ and 2H–NbS$_{2}$ structures. The corresponding phonon dispersion modes of the Nb(Se$_{0.5}$S$_{0.5}$)$_{2}$ structure confirm the dynamical stability. By performing electronic band structure calculations and determining the Fermi surface, we have shown that this material remains metallic, leading to the further investigation of superconductivity. The Eliashberg spectral function and the integration of lambda show that T$_{c}$ is 14.5 K. To achieve higher $T _{c}$, it is important to modify the nature of the chemical bonding. Finally, we suggest that the Nb(Se$_{0.5}$S$_{0.5}$)$_{2}$ structure might be a new class of transition metal dichalcogenide monolayers that, when substituted by S atoms, is likely to superconduct with higher $T _{c}$. This method could be applied well in other materials.

## References

[1] Ge Y, Liu AY (2012). Effect of dimensionality and spin-orbit coupling on charge-density-wave transition in 2$${H}-{Ta}{Se}_{2}$$. Phys. Rev. B.

[2] Bianco R, Monacelli L, Calandra M, Mauri F, Errea I (2020). Weak dimensionality dependence and dominant role of ionic fluctuations in the charge-density-wave transition of $${Nb}{Se}_{2}$$. Phys. Rev. Lett..

[3] Hsu Y-T, Cole WS, Zhang R-X, Sau JD (2020). Inversion-protected higher-order topological superconductivity in monolayer $${\rm wte}_{2}$$. Phys. Rev. Lett..

[4] Tsuppayakorn-aek P, Pungtrakool W, Pinsook U, Bovornratanaraks T (2020). The minimal supercells approach for ab-initio calculation in 2d alloying transition metal dichalcoginides with special quasi-random structure. Mater. Res. Express.

[5] Grüner G (1988). The dynamics of charge-density waves. Rev. Mod. Phys..

[6] Lin D (2020). Patterns and driving forces of dimensionality-dependent charge density waves in 2$${H}$$-type transition metal dichalcogenides. Nat. Commun..

[7] Oh E, Gye G, Yeom HW (2020). Defect-selective charge-density-wave condensation in 2$${H}-{Nb}{Se}_{2}$$. Phys. Rev. Lett..

[8] Petkov V, Chapagain K, Shastri S, Ren Y (2020). Genesis of the periodic lattice distortions in the charge density wave state of 2$${H}-{Ta}{Se}_{2}$$. Phys. Rev. B.

[9] Sipos B (2008). From Mott state to superconductivity in 1$${T}-{Ta}{S}_{2}$$. Nat. Mater..

[10] Kvashnin Y (2020). Coexistence of superconductivity and charge density waves in tantalum disulfide: Experiment and theory. Phys. Rev. Lett..

[11] Lu P (2016). Origin of superconductivity in the Weyl semimetal $${W}{Te}_{2}$$ under pressure. Phys. Rev. B.

[12] Ektarawong A, Tsuppayakorn-aek P, Bovornratanaraks T, Alling B, Kanchanavatee N (2021). Effect of thermally excited lattice vibrations on the thermodynamic stability of tungsten ditellurides $${W}{Te}_{2}$$ under high pressure: A first-principles investigation. Comput. Mater. Sci..

[13] Kiss T (2007). Charge-order-maximized momentum-dependent superconductivity. Nat. Phys..

[14] McMillan WL (1968). Transition temperature of strong-coupled superconductors. Phys. Rev..

[15] Tse JS, Li Z, Uehara K, Ma Y, Ahuja R (2004). Electron-phonon coupling in high-pressure $${Nb}$$. Phys. Rev. B.

[16] Suderow H, Tissen VG, Brison JP, Martínez JL, Vieira S (2005). Pressure induced effects on the fermi surface of superconducting 2$${H}-{Nb}{Se}_{2}$$. Phys. Rev. Lett..

[17] Heil C (2017). Origin of superconductivity and latent charge density wave in $${{NbS}}_{2}$$. Phys. Rev. Lett..

[18] Majumdar A (2020). Interplay of charge density wave and multiband superconductivity in layered quasi-two-dimensional materials: The case of 2$${H}-{Nb}{S}_{2}$$ and 2$${H}-{Nb}{Se}_{2}$$. Phys. Rev. Mater..

[19] Qi Y (2016). Superconductivity in Weyl semimetal candidate $${MoTe}_{2}$$. Nat. Commun..

[20] Paudyal H, Poncé S, Giustino F, Margine ER (2020). Superconducting properties of $${{MoTe}}_{2}$$ from ab initio anisotropic Migdal-Eliashberg theory. Phys. Rev. B.

[21] Mandal M (2018). Enhancement of the superconducting transition temperature by $${Re}$$ doping in Weyl semimetal $${{MoTe}}_{2}$$. Phys. Rev. Mater..

[22] Allen PB, Dynes RC (1975). Transition temperature of strong-coupled superconductors reanalyzed. Phys. Rev. B.

[23] Sanchez JM, Ducastelle F, Gratias D (1984). Generalized cluster description of multicomponent systems. Phys. A Stat. Mech. Appl..

[24] van de Walle A, Asta M, Ceder G (2002). The alloy theoretic automated toolkit: A user guide. Calphad.

[25] Giannozzi P (2009). Quantum espresso: A modular and open-source software project for quantum simulations of materials. J. Phys. Condens. Matter.

[26] Perdew JP, Burke K, Ernzerhof M (1996). Generalized gradient approximation made simple. Phys. Rev. Lett..

[27] Grimme S (2006). Semiempirical $${GGA}$$-type density functional constructed with a long-range dispersion correction. J. Comput. Chem..

[28] Baroni S, de Gironcoli S, Dal Corso A, Giannozzi P (2001). Phonons and related crystal properties from density-functional perturbation theory. Rev. Mod. Phys..

[29] Semenok DV, Kruglov IA, Savkin IA, Kvashnin AG, Oganov AR (2020). On distribution of superconductivity in metal hydrides. Curr. Opin. Solid State Mater. Sci..

[30] Di Cataldo S, von der Linden W, Boeri L (2020). Phase diagram and superconductivity of calcium borohyrides at extreme pressures. Phys. Rev. B.

[31] Deringer VL, Tchougréeff AL, Dronskowski R (2011). Crystal orbital Hamilton population (COHP) analysis as projected from plane-wave basis sets. J. Phys. Chem. A.

[32] Maintz S, Deringer VL, Tchougréeff AL, Dronskowski R (2016). Lobster: A tool to extract chemical bonding from plane-wave based DFT. J. Comput. Chem..

[33] Bovornratanaraks T, Tsuppayakorn-aek P, Luo W, Ahuja R (2019). Ground-state structure of semiconducting and superconducting phases in xenon carbides at high pressure. Sci. Rep..

[34] Tsuppayakorn-aek P, Pinsook U, Luo W, Ahuja R, Bovornratanaraks T (2020). Superconductivity of superhydride $${CeH}_{10}$$ under high pressure. Mater. Res. Express.

[35] Tsuppayakorn-aek P (2020). Route to high-$${T}_{c}$$ superconductivity of $${BC}_{7}$$ via strong bonding of boron-carbon compound at high pressure. Sci. Rep..

[36] Tsuppayakorn-aek P, Phansuke P, Kaewtubtim P, Ahuja R, Bovornratanaraks T (2021). Enthalpy stabilization of superconductivity in an alloying $${S-P-H}$$ system: First-principles cluster expansion study under high pressure. Comput. Mater. Sci..

[37] Revolinsky E, Lautenschlager E, Armitage C (1963). Layer structure superconductor. Solid State Commun..

[38] Heid R, Bohnen K-P, Sklyadneva IY, Chulkov EV (2010). Effect of spin-orbit coupling on the electron–phonon interaction of the superconductors $${Pb}$$ and $${Tl}$$. Phys. Rev. B.

[39] Heikes C (2018). Mechanical control of crystal symmetry and superconductivity in Weyl semimetal $${MoTe}_{2}$$. Phys. Rev. Mater..

[40] Becke AD, Edgecombe KE (1990). A simple measure of electron localization in atomic and molecular systems. J. Chem. Phys..

[41] Tsuppayakorn-aek P, Luo W, Watcharatharapong T, Ahuja R, Bovornratanaraks T (2018). Structural prediction of host-guest structure in lithium at high pressure. Sci. Rep..

[42] Tsuppayakorn-aek P (2018). The ideal commensurate value of $${Sc}$$ and the superconducting phase under high pressure. J. Appl. Phys..

[43] Kotmool K (2020). Structural phase transitions, electronic properties, and hardness of $${RuB}_{4}$$ under high pressure in comparison with $${FeB}_{4}$$ and $${OsB}_{4}$$. J. Phys. Chem. C.

[44] Tsuppayakorn-Aek P, Sukmas W, Ahuja R, Luo W, Bovornratanaraks T (2021). Stabilization and electronic topological transition of hydrogen-rich metal $${Li_{5}MoH_{11}}$$ under high pressures from first-principles predictions. Sci. Rep..

[45] Momma K, Izumi F (2008). VESTA: A three-dimensional visualization system for electronic and structural analysis. J. Appl. Crystallogr..

[46] Kokalj A (1999). Xcrysden—A new program for displaying crystalline structures and electron densities. J. Mol. Graph. Modell..

